# Supplementary material for: Base excision repair and double strand break repair cooperate to modulate the formation of unrepaired double strand breaks in mouse brain
Source: Nat Commun. 2024 Sep 4;15:7726. doi: 10.1038/s41467-024-51906-5 (PMC11375129; doi:10.1038/s41467-024-51906-5)
Supplement: Supplementary file 1 — Supplementary Information [file 41467_2024_51906_MOESM1_ESM.pdf]

Base excision repair and double strand break repair cooperate to modulate the formation of unrepaired double strand breaks in mouse brain

Aris A Polyzos[1*], Ana Cheong[2], Jung Hyun Yoo[1], Lana Blagec[1], Sneh M Toprani[2], Zachary D Nagel[2] and Cynthia T McMurray[1*]

[1]Division of Molecular Biophysics and Integrated Bioimaging,

Lawrence Berkeley National Laboratory,

Berkeley, CA 94720.

[2]Department of Environmental Health

John B Little Centre of Radiation Sciences

Harvard T.H. Chan School of Public Health

Boston, MA 02115.

*To whom correspondence should be addressed:

Email: ctmcmurray@lbl.gov or aapolyzos@lbl.gov

Methods

Our research complies with relevant ethical regulations.

Animals

The Institutional Animal Care and Use Committee approved all procedures. Animals were treated under guidelines for the ethical treatment of animals and approved by IACUC protocol #274005 at Lawrence Berkeley Laboratory. All animal work was conducted according to national and international guidelines. Male C*57BL/6J* were used in every experiment and referred to as C*57BL/6J* or wildtype (WT) mice (Jackson Labs cat#000664). The Male C*57BL/6J* were used as controls in an ongoing study using a disease model where males were used to keep gender consistency. At least 3 animals (n=3) were used in each tissue experiment.

Brain Tissue Lysate Preparation

Dissected brain tissues were flash frozen in liquid nitrogen and were preserved at -80ºC until lysate preparation. Brain tissue was placed in a microcentrifuge tube and thawed on ice for approximately 10 min before adding lysis buffer consisting of T-PER Tissue Protein Extraction Reagent (Thermo Scientific, Cat No 78510), ~8.7x Halt protease inhibitor cocktail (from 100x, Thermo Scientific, Cat No 78430), ~4.3x Halt phosphatase inhibitor cocktail (from 100x, Thermo Scientific, Cat No 78420). After adding the lysis buffer, the tissue sat on ice for 2-3 min before being triturated with a Bel-Art extended handle pestle (Millipore-Sigma, Cat No BAF199210001). The triturated tissue sample was centrifuged at 16,100g, 4 ºC for 1.5 min in Eppendorf 5425R refrigerated microcentrifuge. Following the centrifugation, the sample was subject to 4 cycles of pulse sonication on ice, performed in which 2 cycles of 10s-on and 30s-off followed by centrifugation at 16,100g, 4 ºC for 1.5 min, 1 cycle of 10s-on and 30s-off followed by centrifugation at 16,100g, 4 ºC for 1.5 min, and then 1 cycle of 10s-on and 30s-off. Sonication was performed

using Branson Sonifier Cell Disruptor 185. The resulting homogenate was centrifuged once more at 16,100g, 4 ºC for 30 min and supernatant was used for the protein quantification assay, SDS-PAGE and western blotting analyses.

Protein Quantification Assay

The protein concentration of each lysate was quantified using Pierce 660 nm Protein Assay reagent (Thermo Scientific, Cat No 22660). Briefly, a small volume of each lysate was diluted 1:10 in phosphate-buffered saline (PBS). 10 µL of diluted lysate was mixed with 340 µL of Pierce 660 nm Protein Assay reagent in a 1.7 mL microcentrifuge tube and then incubated at room temperature (RT) for approximately 5 min before loading 150 µL of each mixture in each of 2 wells of a flat bottom, transparent 96-well plate. For each lysate, a mixture was prepared and analyzed in triplicate by Infinite M1000 microplate reader (Tecan) using Tecan i-control software.

SDS-PAGE and Western Blot

SDS-PAGE samples were prepared by mixing brain tissue lysate containing 60 µg total protein (20 µg for apurinic/apyrimidinic endonuclease 1 or APE1, and 10 µg for glyceraldehyde 3-phosphate dehydrogenase or GAPDH), T-PER Tissue Protein Extraction Reagent (to level the volume of all the samples), 1x NuPAGE LDS Sample Buffer (from 4x, Invitrogen, Cat No NP0007) and 1x NuPAGE Sample Reducing Agent (from 10x, Invitrogen, Cat No NP0009). Each sample was boiled at ~95 ºC for 10 min and centrifuged at 15,000g, at RT for 5 min. Total proteins in each SDS-PAGE sample were subsequently resolved along a Novex WedgeWell Tris-Glycine 4-12 % Mini gel (Thermo Fisher, Cat No XP04205BOX) in XCell SureLock Mini-cell Electrophoresis system (Thermo Fisher). The resolved proteins were transferred to a nitrocellulose membrane using Trans-Blot Turbo Transfer System (Bio-Rad), with a standard protocol (25 V, 1.0 A, 30 min). The resulting nitrocellulose membrane was blocked in 5% non-fat dry milk in PBS + 0.05 %

Tween-20 (PBST) for 1 hour at RT. The membrane was then incubated in 5 % non-fat dry milk in PBST + primary antibody for 1 hour at RT. The membrane was then washed 3 times with PBST. The membrane was subsequently incubated in 5 % non-fat dry milk in PBST + secondary antibody for 30 min at RT. The membrane was next washed 3 times with PBST and once with PBS. Lastly, Amersham ECL Select Western Blotting Reagent (Sigma-Aldrich, Cat No GERPN2235) was applied to the membrane and the western blot image was developed using VersaDoc MP 4000 Imaging System (Bio-Rad) and Quantity One 1-D Analysis software (Bio-Rad). Quantification of protein band intensity was performed using Image Lab software (Bio-Rad). The specific antibodies used in the analysis are listed in key resources Supplementary Table 2 and antibody testing is provided in Supplementary Table 3.

Brain Tissue Sections

We collected brains from male mice (7-10 wks and 70-90 wks of age) of *C57BL6/J* mice. Brains were cut into 4 coronal sections and arranged in a holder filled with OCT (Tissue-Tek O.C.T. from Sakura), and immediately frozen in isopentane bath cooled by liquid nitrogen, prior to storage at -80°C. This arrangement of the tissue permitted concurrent cutting of all 4 sections at a time. Cuts were made so that all the relevant regions (caudoputamen of striatum, CA1 region of hippocampus, the granular and molecular layer of Crus1 of the cerebellum, and the entorhinal area of the cortex) were present in each cut. Sectioning onto slides (Histobond from VWR) was performed on a cryostat (Leica CM1950) using cut settings (chuck = -14°C, blade = -15°C) and cutting 10-15μm thick sections. Slides were air dried (15 min) and stored at -80°C until use. In parallel, the dissected tissue of equal weight before freezing was dispersed for cell number counting on a hemocytometer.

Immunofluorescence Staining and Imaging

Primary antibodies used were mouse anti-NeuN Alexa-488 conjugate (Millipore #MAB377X) (1:500), mouse anti-GFAP Cy3 conjugate (Abcam #ab49874) (1:500), mouse anti-APE1 (Novus #13B8E5C2) (1:500), mouse anti-Ku80 (Santa Cruz #515736) (1:500), mouse anti-ERCC1 (Santa Cruz #17809) (1:500), rabbit anti-MSH2 (Abcam #92473) (1:500), mouse anti-MSH3 (Millipore #MABE324) (1:500), rabbit anti-MSH6 (Abcam #ab92471) (1:500), rabbit anti-XPA (AbClonal #A1626) (1:500) and rabbit anti-MRE11 (Novus #NB100-142) (1:500). (**Table S1**) Secondary antibodies used were donkey anti-mouse Alexa-488 (Jackson #715-545-150), goat anti-mouse Alexa-568 (Invitrogen #A21124), donkey anti-rabbit Alexa-488 (Jackson #711-545-152), and goat anti-rabbit Alexa-555 (Invitrogen #A32732). Anti-mouse antibodies were tested and selected for those having the least amount of background staining, which was typically visible as staining of blood vessels amongst various commercially available options.

Brain sections on slides were thawed and fixed with 4% PFA for 20min at 4°C, then washed once with PBS. They were then pre-extracted with RNase in CSK buffer, i.e., 0.3mg/mL RNase A (New England Biolabs) in (10mM PIPES pH 7.0, 100mM NaCl, 300mM sucrose, 3mM $MgCl_2$, 0.7% Triton X-100). Lipofuscin autofluorescence was quenched by soaking in 1x TrueBlack (CellSignal #92401) in 70% EtOH (30sec) (care was taken not to allow sections to dry out), prior to 3 washes in PBS. Sections were then blocked with Fc Receptor Blocker (Innovex #NB309) for 15min at RT and then with Background Buster (Innovex #NB306-50) for 15min at RT, prior to washing once with PBS. Sections were then coated with 200µL of primary antibodies (1:500 diluted 1:500 in 10% Background Buster: PBS) and incubated for 1hr at 37°C or overnight at 4°C, prior to 2 washes of 10min each with PBS. Sections were then stained with 200µL of secondary antibodies (1:1,000 diluted in 10% Background Buster: PBS) and DAPI (10µg/mL) for 30min at 37°C. Finally, the slides were washed 2 times with PBS, 15min each, and refixed with 4% PFA for 10min. Sections were mounted using Immu-Mount (Epredia) and #1 coverslips (Electron Microscopy Services), sealed with nail polish, and stored at -20°C until they could be imaged.

IF measurement of DSBs in tissues

Methods are the same as for IF detection of proteins except an antibody for γH2AX[49] or 53BP1[50,51] was used (Table S1). Tissue sections were co-stained with DAPI, an antibody to NeuN, and antibodies to γH2AX. DSBs in neurons were detected as co-staining of γH2AX and NeuN. Quantification of IF staining intensity for γH2AX was determined from 50 randomly selected cells of each type within the tissue sections or in cells.

Quantification of DSBs by CometChip assay in cells from tissue

Samples of young and old mouse brain regions (CBL, CTX, STR, HIP) were prepared before use for the Comet assay. A young (15wk) and an old (100wk) C57BL/6 mouse were sacrificed and 2-3 mm samples of the four brain regions of interest were collected, flash frozen and stored in liquid nitrogen until use. Samples were minced with scissors in 30 µL of buffer (HBSS, 20 mM EDTA, 10% DMSO) on ice. Next, 400 µL of buffer was added (200 µL for the HIP region, as it usually has the least cells). The mix was added on a 40µm filter-top tube and centrifuged for 3 min at 150 g and 4°C. The cell concentration for each region was estimated by mixing 10 µL of each sample with 1 µL of 100x SYBR Gold (Invitrogen, Cat no. S11494), pipetting 10 µL on a hemocytometer (Hausser Scientific, PA, USA) secured on a glass slide and counting cells at 5X magnification using an inverted LED fluorescence motorized microscope (Zeiss LSM 710 microscope, Carl Zeiss Microscopy, GmbH, Germany). Every sample was then brought to 300,000 cells/mL in 25 µL with PBS and used for the Comet assay, as described for glial cultures.

Brain sample preparation for the tissue Comet assays

Samples of young and old mouse brain regions (CBL, CTX, STR, HIP) were prepared before use for the Comet assay. Young (15 wks) and old (roughly 100 wks) C57BL/6 mice were sacrificed

and 2-3 mm samples of the four brain regions of interest were collected, flash frozen and stored in liquid nitrogen until use. Samples were minced with scissors in 30 µL of buffer (HBSS, 20 mM EDTA, 10% DMSO) on ice. Next, 400 µL of buffer was added (200 µL for the HIP region, as it usually has the least cells). The mix was added on a 40 µm filter-top tube and centrifuged for 3 min at 150 g and 4ºC. The cell concentration for each region was estimated by mixing 10 µL of each sample with 1 µL of 100x SYBR Gold (Invitrogen, Cat no. S11494), pipetting 10 µL on a hemocytometer (Hausser Scientific, PA, USA) secured on a glass slide and counting cells at 5X magnification using an inverted LED fluorescence motorized microscope (Zeiss LSM 710 microscope, Carl Zeiss Microscopy, GmbH, Germany). Every sample was brought to 300,000 cells/mL in 25 µL with PBS and used for the Comet assay.

Quantification of DNA damage using the Comet assay

Alkaline and neutral Comet assays were performed to quantify the amount of DNA damage and DSB in different young or old mouse brain regions (CBL, CTX, STR, HIP). In brief, 25 µL of each sample at 300,000 cells/mL was mixed with 250 µL molten low-melting agarose (R&D Systems, Cat no. 4250-050-02). 40 µL of this mixture was pipetted onto a 3-well FLARE slide (R&D Systems, Cat no. 3950-075-02) and spread with the pipette tip. Slides were placed in the dark for 10 min at 4ºC, and then immersed in Lysis Solution (R&D Systems, Cat no. 4250-010-01) overnight at 4ºC. For the Neutral Comet, slides were immersed in cool neutral buffer (0.1 M Tris, 0.5 M Sodium Acetate, pH 9) for 30 min, before electrophoresis was performed at 21 V for 45 min at 4ºC in 850 mL of neutral buffer. Slides were put in DNA Precipitation Solution (1 M Ammonium Acetate in 95% EtOH), then 70% EtOH for 30 min each. For the Alkaline Comet, slides were immersed in the alkaline buffer (200 mM NaOH, 1 mM EDTA, pH>13) for 20 min in the dark, before electrophoresis was performed at 21 V for 30 min at 4ºC in 850 mL of alkaline buffer. Slides were put twice in distilled water and once in 70% EtOH for 5 min each. For both Comet assays,

slides were dried at 37ºC for 10-15 min. 100 µL 1x SYBR Gold (Invitrogen, Cat no. S11494) were placed onto each well for 30 min in the dark, before being rinsed with distilled water. Slides were dried completely at 37ºC, and fluorescence 5x5 tilescan (0.6 zoom) images of the comets were captured at 5X magnification using an inverted LED fluorescence motorized microscope (ZEN 2.1 SP3 FP3 (black) Zeiss LSM 710 microscope, Carl Zeiss Microscopy, GmbH, Germany). Comet images were analyzed using Trevigen comet analysis software (R&D Systems, MN, USA). We scored at least 200 cells per sample. The average value of DNA in the comet tail (%) and tail moment were used as the parameters for estimating basal DNA damage and DSB levels using alkaline and neutral Comet assays, respectively.

Preparation and culturing of primary glia from brain regions

Intact brains were collected from day 1-3 newborn pups (P1-3) of male C57BL/6J mice. Brain regions (hippocampus, cerebellum, cortex, and striatum) pooled from 4-8 pups were isolated in a solution of HBSS supplemented with 1mM L-glutamine, 1mM sodium pyruvate and 1x Non-Essential Amino Acids. These tissue suspensions were digested in 5 mL 0.025% Trypsin-EDTA (Gibco 25300056) for 20 min at 37˚C with gentle rocking. Tissue pieces were pelleted (5 min, 300g, room temperature (RT)) and then gently triturated 20-30 times in pre-warmed astrocyte media (Neurobasal A base media (Thermo-Fisher #10888022), 10% FBS (JRS 43635), 2% B27 Supplement (Themo-Fisher #1504044), 25mM glucose, 2mM sodium pyruvate, 2mM GlutaMax, 1x non-essential amino acids (Quality Biologicals 116-078-721EA), 1x antibiotic/antimycotic (Gibco #15240062) using a 5 mL pipet, to dissociate into cells. Each cell suspension was tested for mycoplasma and negative cultures were passaged 3 times to expand cell number. Each passage was cultured for 6-10 days (at 37˚C, 5% CO_2) with media exchanges every 2-3 days. At the end of three passages (roughly 25 days in culture), some cultures developed traces of mycoplasma but the transfection results of these cultures did not differ significantly from the

negative cultures. Astrocyte cell purity and homogeneity was established by immunofluorescent analysis using anti-GFAP antibody-Cy3 conjugate (Abcam ab49874).

Quantification of CometChip assays for glial cells

Neutral CometChip assays were performed to quantify the amount of DSB in the primary glia isolated from different mouse brain regions (CBL, CTX, STR, HIP). Assays were performed in three different biological replicates (on three different days from the same batch of cells used for FM-HCR assays). The CometChip preparation and protocol were carried out as described[52,85-87]. All chemical reagents used for CometChip assays were obtained from Sigma or VWR unless otherwise stated. In brief, 10,000 cells per 100 µL/well were loaded for each cell type in 96-well plates for 60 min at 37°C and 5% CO_2. After settling by gravity, excess cells were aspirated, and the chips were rinsed with 1X DPBS (Gibco, Cat no.14-040-133). Then, 1% (w/v) molten low-melting agarose (Gold Biotechnology, Cat no. A-204-25) in 1X DPBS was overlaid onto the chip and allowed to gelate for 2 min at room temperature followed by 2 min at 4°C. The neutral chip was submerged overnight at 43°C in pre-warmed neutral lysis solution (2.5 M NaCl, 100 mM Na_2EDTA, 10 mM Tris, 1% N-lauroyl sarcosine, pH 9.5 (TCI America, Cat no. S05975G) with 0.5% Triton X-100 and 10% DMSO). After overnight incubation, for the neutral comet chip, DNA unwinding was performed with neutral buffer (2mM Na_2EDTA, 90mM Tri's base and 90mM boric acid in distilled water) at 4°C for 60 min. Electrophoresis was carried out in same DNA unwinding buffer at 4 °C for 60 min at constant voltage of 0.6 V/cm and 6 mA for the neutral chip. Following the electrophoresis, the chips were neutralized using neutralization buffer [0.4 M Tris-HCl buffer, pH 7.5] and stained using 1X (0.01% (v/v) of 10,000X stock) SYBR gold nucleic acid gel stain (Invitrogen, Cat no S11494) for 15 min at room temperature. Fluorescence images of the comets were captured at 10X magnification using an inverted LED fluorescence motorized microscope (Zen 3.2 pro blue edition, Zeiss Apotome 2, Carl Zeiss microscopy, GmbH). Comet images were

analyzed using Trevigen comet analysis software (R&D systems, MN, USA). At least 50 cells were scored per biological replicate. The median tail length (μm) was used as the parameter for estimating basal DSB levels in the neutral CometChip assay [52,53,85-87].

Transfections in primary glia cultures

Primary mouse glia was used live or thawed and cultured in prewarmed DMEM high glucose medium supplemented with 20% fetal bovine serum, GlutaMax, non-essential amino acids, plasmocin, normocure, and penicillin/streptomycin. For transfection experiments, 0.05 million cells were seeded per well in 12-well plates and allowed to attach overnight. All cells were passages once to achieve about 80% confluency. On the day of transfection, cell culture medium was changed to 1 ml OPTI-MEM, and the cells were transfected with 1 µg plasmid cocktails using lipofectamine 3000 (Thermofisher Scientific) according to the manufacturer's protocol. The transfection medium was changed to cell culture medium at 4 hours post transfection. At 24 hours post transfection, the cells were washed with PBS, trypsinized, and filtered through a 40 µm strainer cap tubes for flow cytometry analyses.

FM-HCR Reporter vectors

All reporter plasmids used for FM-HCR were pMax vector-based. They were engineered with site-specific DNA lesions as previously described[55-57]. For NHEJ reporter, pMax BFP with a ScaI recognition site (BFP-ScaI) was linearized with ScaI restriction and purified using phenol-chloroform extraction and ethanol precipitation method. For the NER reporter, pMax mPlum was irradiated with UV-C light at 800 J/m^2 and purified using ethanol precipitation method. The mPlum UV reporter was validated by analytical digest with T4PDG enzyme. For BER, the pMax GFP THF reporter was used to report long-patch base excision repair activity, and, for MMR, the pMax GFP A:C MMR reporter was generated using the described protocol[66,68]. Briefly, the base plasmid

pMax-GFP C289T was nicked with Nt. BspQ1, digested with ExoIII to generate ssDNA, annealed with an oligo, that contains the mismatch. Subsequently the oligonucleotide was extended by an overnight incubation with DNA polymerase and ligase, cleaned up with T5Exo digestion, precipitated with PEG8000 solution, and purified using phenol-chloroform extraction and ethanol precipitation. The GFP AC MMR reporter was validated in TK6 MSH2$^{-/-}$ and WT cells.

HR activity was reported using a new BFP HR assay, which was designed with reference to the reported pCX-NNX-GFP HR assay[85]. Briefly, three gene blocks were designed to generate a wild-type plasmid (2Nt-BFP), a donor plasmid (2Nt-D3BFP), and a plasmid (2Nt-D5BFP) with a ScaI restriction recognition site. Each gene block contains a NheI recognition site at the 5' end a SacI recognition site were deleted at the 3'end. Adjacent to the NheI recognition site are two recognitions sites for Nt. BspQ1 (2Nt) that flank the recognition sites for MluI and ScaI, and all of which are upstream of the Kozak consensus sequence. For 2Nt-D5BFP, the last four base pairs of the Kozak sequence and the first 19 base pair from the 5' end of BFP were deleted to generate a novel Pst1 recognition site and a truncated BFP sequence that renders the protein encoded by the plasmid non-fluorescent. For 2Nt-D3BFP, 99 base pairs were deleted from the 3' end to generate a truncated BFP protein that renders the protein expressed by the plasmid non-fluorescent. The 2Nt-BFP, which serves as the positive control, is engineered to have a full length BFP sequence, and thus is fluorescent. To generate the three HR reporters, pMax vector and gene blocks were digested with NheI and SacI and purified using gel extraction (Monarch DNA gel extraction kit, NEB) for the linearized vector and column purification (Monarch PCR and DNA cleanup kit, NEB) for the gene blocks. The gene blocks were then cloned into the linearized pMax vectors and amplified in DH5α competent cells. Putative positive clones were selected by kanamycin resistance and validated by sequencing using pMax reverse primer. Individual reporters were amplified in 1L LB medium with kanamycin and extracted using PureLink endotoxin-free giga plasmid purification kit (Thermofisher Scientific). For generating the linearized

2Nt-D5BFP for HR assay, 2Nt-D5BFP was linearized with PstI and purified using phenol-chloroform extraction and ethanol precipitation method. Fluorescence of each vector was tested in cell lines using flow cytometry analyses and the recombinogenic events between the linearized 2Nt-D5BFP and 2Nt-D3BFP were validated in HR deficient cell lines.

FM-HCR Reporter cocktails

Three reporter cocktails were prepared for FM-HCR analyses. Damaged reporter cocktail A is composed of 100ng of PstI-linearized 2Nt-D5BFP (HR), 100ng of pMax GFP THF (BER), and 100ng of pMax mPlum as transfection control. Damaged reporter cocktail C is composed of 100ng of GFP_AC reporter (MMR) and 100ng of pMax_ mPlum as transfection control. A final amount of 2Nt-D3BFP was added to each damaged plasmid cocktail so a total of 1400 ng plasmid DNA was used for transfection. A single undamaged reporter cocktail is composed of 1100ng of 2Nt-D3BFP, 100ng of each of BFP_ScaI, pMax_GFP, and pMax_mPlum.

FM-HCR Analysis

Primary glia were transfected with sufficient efficiency (4-8%) to afford a robust analysis of DNA repair capacity by flow cytometry analyses. All transfected cells were analyzed by flow cytometry (Attune NXT Flow cytometer, Thermofisher Scientific) at 24-hours post transfection. Data analyses were performed as previously described[55-57] to determine the DNA repair capacity of the primary glia. Briefly, for each transfection (n=5), the product of the fluorescent positive cell counts and mean fluorescence intensity of the positive cells in each gate was normalized to that in the gate for transfection control. Activity of each DNA repair pathway was reported as % reporter expression, which is the ratio between the normalized reporter expression in the damaged and the undamaged cocktail multiplied by 100. The activity level of each DNA repair pathway studied is positively correlated with the % reporter expression. FM-HCR assays report independent repair

mechanisms and absolute reporter expression depends on factors specific to each assay precluding statistical comparisons between pathways. Thus, % reporter expression approximates the percentage of reporter plasmids that have been repaired by the pathway of interest, this approach provides a rough estimate of the relative activity of multiple pathways based on this metric. FM-HCR assays report independent repair mechanisms and absolute reporter expression and depend on factors specific to each assay, precluding statistical comparisons between pathways. The FM-HCR assays are highly validated, robust reporters of DNA repair activity of cells and reflected activity of the line. For example, using the same FM-HCR assays, a panel of human lymphoblastoid cell lines from apparently healthy individuals consistently yielded around 2% reporter expression for HR, as was observed in the glia, but had greater than 30% reporter expression for NHEJ[55-57]. At the same time, expression for the BER reporter was high (~40%) in mouse brain glial cells and in human lymphoblastoid cell lines[55-57].

DNA Oxidation Assay

Three male *C57BL/6J* mice were assessed from both young (7-10wks old) and old (70-90wks old) ages, as indicated, referred to as wildtype (WT) mice (Jackson Labs cat#000664). Briefly, the CBL, STR, HIP, and CTX were collected from each animal and the DNA was isolated from 25 mg of tissue using DNeasy Blood and Tissue Isolation kit (according to the manufacturer's protocol). The DNA was quantified (using a Nanodrop instrument, Thermo-Fisher) and 25-30 µg DNA was diluted in 45µL distilled/deionized water. The genomic DNA was sheared at low power using a sonicator for 30sec on ice. Double stranded DNA was heat denatured at 95°C for 5 min before being snap cooled on ice for 10min. DNA was then digested with 150U of Nuclease P1 (New England Biolabs) in 1x P1 buffer (New England Biolabs) for 30min at 37°C. The reaction was stopped by heat inactivating for 10min at 70°C and addition of 1 µL 1M Tris-Cl pH 8.0. The nucleotide solution was further digested with 5U Quick CIP Calf Alkaline Phosphatase (New

England Biolabs) for 30 min at 37°C, before being heat inactivated for 5 min at 80°C and snap cooled on ice until it was used in the assay. Oxidized DNA/RNA was then quantified following manufacturer's protocol (DNA/RNA Oxidative Damage ELISA Kit, Cayman Chemicals #589320).

XJB-5-131 Treatment

XJB-5-131 (gift form P Wipf (University of Pittsburgh) was synthesized[61] and treatment protocols were as previously descriend[59-62]. Lyophilized, powdered XJB-5-131 was reconstituted in DMSO at a concentration of 1 µg/µL. These samples were aliquoted and kept at -80°C. On the day of injection, the XJB-5-131 solution was mixed with 0.2 µm filtered and pre-warmed PBS (100°C) to reach a final concentration of 2 mg/kg mouse body weight in 200 µL solution. This was heated for 10 sec and the solution (200 µL) was injected, within 30min of preparation, by intraperitoneal injection (IP). Administration started at 60 weeks of age and continued three times per week for the length of the study. Vehicle treatments were identical except that XJB-5-131 was replaced by filtered PBS.

Peroxide treatment in cells

Mouse Embryonic Fibroblast cell line (NIH3T3, ATCC Cat# CRL-658) were grown overnight in 8 well microwell slides (Ibidi Cat# 80826). Experiment 1 cultures in Fig. 9 were peroxide treated (100µM) with and without XJB-5-131 (200µM) for 30 minutes. After all treatments, the cells were washed briefly with PBS and fixed in 4% Paraformaldehyde for 15min prior to immunofluorescent staining.

Transfection and targeting of Cas9 and Cas9D10A

Cells were transfected with plasmids expressing the hCas9-D10A nickase or Cas9. DNA using Lipofectamine 3000 (ThermoFisher Cat#L3000001) following the manufacturers protocol. Briefly,

DNA (500ng total per well) was added along with 0.5µL P3000 reagent and 0.5µL Lipofectamine 3000 reagent after a short (10min) mixing and pre-incubation. The transfected DNA was a combination of guide RNA plasmid (recognition sequence 5' CCATATTCCACGTCCTACAG 3'), hCas9D10A nickase (2) (Addgene Cat#41816) or Cas9 (pCDNA3.3TOPO-T7-hCas9) (Addgene Cat #161876) in the presence of Doxycyclin (2µg/mL), with or without XJB-5-131 (200µM). After 6 hours, they were washed with PBS and fixed in 4% Paraformaldehyde for 15min prior to immunofluorescent staining.

Statistical analysis

Statistical analysis is reported as appropriate in each Figure. For all box and whisker plots, the boxes represent 50% of all data points with the line indicating the median value. The remaining 50% of datapoints are in the whiskers, distributed as the 25% maximal values above the box and 25% minimal values below the box. For CometChip assays, graphical representations were expressed as Mean ± SD and one-way ANOVA followed by Tukey's multiple comparisons test was performed using GraphPad PRISM version 9.5.1 for Mac, GraphPad Software, San Diego, California USA. $P<0.05$ was considered as statistically significant using ANOVA models with post-*hoc* multiple comparison tests by GraphPad PRISM (GraphPad Software, LLC).

Data Availability

There are no restrictions on the availability of these data. Source data are provided in Source Data file NCOMMS-23-38584B with this paper for plots reporting means/averages in bar charts and tables. Reagent used are presented in Supplementary Table 2. Antibody testing results are presented in Supplementary Table 3. Uncropped gel images are provided in Supplementary Source Data file NCOMMS-23-38584C

Supplementary Figure Legends

Supplementary Fig. 1: The machinery to carry out DNA repair is expressed in the mouse brain.

Expression of DNA repair machinery was determined from four brain regions: the Cerebellum (CBL), Striatum (STR), Cortex (CTX) and Hippocampus (HIP) of 10-11 wks (a) and 75 wks (b) in C57BL/6J male mice. The C57BL/6J strain is clonal, and the animals have a genetically identical protein profile. We selected 4 genetically identical animals from the colony, n=2 young animals of 10 wks and n=2 animals of 75 wks. At each age, the extracts from the two each animal were run side by side, indicated by the number 1 and 2 in the gel. Collectively, eight technical replicates of the SDS-PAGE gels were analyzed. Five technical replicate sets of SDS-PAGE gels were analyzed in Fig.1 and the three additional replicate sets included in Supplementary Fig. 1. Each replicate gel was transferred to membranes and probed with specific antibodies to a representative protein (P) or GAPDH (C), shown to the side of each plot. Representative proteins detected by western blot are Xeroderma pigmentosum complementation group A (XPA) of the NER/TCR pathway, the MutS homolog 2 (MSH2) of mismatch repair (MMR) pathway, and X-Ray Repair Cross Complementing 5 (Ku80 subunit) for non-homologous end-joining (NHEJ) pathway. Protein antibodies are listed in Supplementary Table 2. The expression of DNA repair proteins in each region was plotted relative to the CBL. Error bars represent the minimum (upper bar) and maximum (upper bar) values for the two clonal samples (n=2) at each age. Full uncropped gels are provided in Supplementary Source File NCOMMS-23-38584B. Optimization testing for the antibodies used is provided in Supplementary Table 3 of the Supplementary Source File NCOMMS-23-38584C.

Supplementary Fig. 2: DNA repair machinery is cell type specific.

(a, b) Magnified IF images of cells cell type distribution of MSH6 in four brain regions (CBL, STR, CTX, and HIP) in brain tissue of 10wk C57BL/6J male mice. (a) (NeuN)(+) cells are neurons and (b) NeuN(-) cells are glia. Each brain section was co-stained with DAPI (D, blue), NeuN neuronal marker (N, purple), and MSH6 (M, green), shown together as a series of individual images or as an overlay of all three on the far left (D/N/M). (a) MSH6 expression co-stains in NeuN(+) neurons in four brain regions; and (b) staining is weak in NeuN(-) glia of all four brain regions. Scale bar is 10µm. (c, d) IF intensity of APE1, XPA, MRE11, Ku80 and MSH6 expression in three NeuN(+) and three NeuN(-) cells in the CTX of a 7wk mouse brain. The same cell type relationship holds for all pathway proteins: expression is prominent in (c) NeuN(+) neurons and weak in (d) NeuN(-) glia. The light blue contours in the glial cells indicate the position of the nucleus and highlights the poor protein staining intensity. Numbers 1-3 indicate neuron and glial cell in same field. Scale bar is 10µm.

Supplementary Fig. 3: Single cell quantification of DNA repair machinery expression in neurons and glia of 75wk mouse brain sections.

The single cell type analysis of DNA repair machinery expression in the brains of in C57BL/6J male mice at 75wk animals. Quantified were APE1, XPA, MRE11, Ku70, and MSH6, as indicated. Expression and analysis are described in Fig. 3. The average per cell expression was quantified from 50 NeuN(+) neurons and 50 NeuN(-) glia, randomly selected in four brain regions: CBL (blue), STR (green), CTX (pink), HIP (orange) in tissue section from n=3 animals. Data are displayed as a box and whisker plot, where a box indicating the 25th to 75th percentile values, the line indicates the median, with the lower whiskers representing the minimum 25% of data values and the whiskers above the box representing the 25% maximum values. Statistical probability of variance (*P*) calculated in n = 50 cells of each type (Neu) Neuron and (GL) Glia using 2-tailed

homoscedastic t-test. Statistical significance among regional expression is tabulated in Supplementary Table 1. *p = <.05, **P<0.005, ***P< 0.0005, ****P<0.00005.

Supplementary Fig. 4: The average Neuron:Glia (N/G) ratio of expressed DNA repair machinery.

A comparison of DNA repair protein expression ratio in neurons versus glia (Ratio N/G) for APE1 (BER), XPA(NER), MRE11 (HR) Ku80m(NHEJ) and MSH6 (MMR) was determined from median of the box and whisker plot for protein expression in neurons divided by the median of the box and whisker plot for protein expression in glia in 7wk animals (taken from Fig. 3). The brain regions, repair proteins and DNA repair pathways are indicated; CBL (blue), STR (green), CTX (pink), HIP (orange).

Supplementary Fig. 5: Quantification of the neutral comet for DSBs in tissue cells.

Analysis of DSBs in the dispersed brain cells of in n=4 C57BL/6J male mice at 7 and 75 wks by neutral comet assay. (a) Comet tails from commercial standards provided by R&D Systems, Minneapolis, MN, USA). Internal standards are immortalized human lymphoblasts treated with low, medium, or high concentrations of etoposide (standards are proprietary). (b) Quantification of (n>700) neutral comet tails per standard sample displayed as % Comet Tail, Tail Length or Comet Tail Moment (% Tail DNA * Tail Length /100). The probability statistics were derived from a 1-way ANOVA **** P < 0.00001 for all cell type comparisons. (c) Images of neutral comets for dispersed brain cells the CBL, STR, CTX, and HIP of C57BL/6J male mice (n=4) at 7 and 75 wks mice. (d) Quantification of (n>700) neutral comet tails in mouse brain cells at early ages (7wks, light gray) and (d) older ages (75wks, dark gray) displayed as % Comet Tail, Tail Length or Comet

Tail Moment (% Tail DNA * Tail Length /100), as indicated. The probability statistics were derived from a 1-way ANOVA **** P < 0.00001 for all regional comparisons.

Supplementary Fig. 6: Primary glia in culture reflect features of glia in tissues.

(a) Characteristic images of cultured primary glial cells from CBL, STR, HIP, and CTX stained positively with markers of glia (glila fibrillary acidic) (GFAP, red) and nuclear DAPI staining (blue). Percentage of GFAP positive cells per culture, at 3rd passage after isolation is (CBL, 90.2 ± 6.1%), (STR, 80.5 ± 11.5%), (CTX, 81.4 ± 11.5%), (HIP, 74.3 ± 14.9%). (b) Images of nuclear staining of MSH6 (red) in cultures of primary glia isolated per brain regions (CTX, CBL, STR, HIP) at postnatal day 2 (P2) from n=4 C57BL/6J mice. Light blues circles represent the nuclear periphery; red indicates nuclear staining of anti-MSH6 antibody. The 10µm scale bar is shown. (c, d) Quantification of IF intensity from MSH6 in (c) primary cultured glial cells and (d) in tissue sections of different brain regions of C57BL/6J male mice at 7 wks (n=3 animals). Data are displayed as a box and whisker plot, where a box indicating the 25th to 75th percentile values, the line indicates the median, with the lower whiskers representing the minimum 25% of data values and the whiskers above the box representing the 25% maximum values. Statistical probability of variance (*P*) in n=3 cultures was calculated from quantifying n = 50 cells/culture of each type using a 1-way ANOVA *p<.01, **p<.001, ***p<.0001, ****p<.00001. (e.f) Same as (c, d), but for Ku80. The results from brain regions are indicated as CBL (blue), STR (green), CTX (pink), HIP (orange).

Supplementary Fig. 7: DSB formation exceeds the repair capacity of DSBR machinery.

Side by side comparison between (a) the expression level of Ku80 and (b) the level of γH2AX DSB marker in neurons among brain regions of young (7wks) male mice. (a) The IF quantification of the Ku80 varies in four brain regions taken from Fig. 3. Data are displayed as a box and whisker plot, where a box indicating the 25th to 75th percentile values, the line indicates the median, with the lower whiskers representing the minimum 25% of data values and the whiskers above the box representing the 25% maximum values. The probability statistics were derived from a 1-way ANOVA, **** $P < 0.00001$ for all regional comparisons. (b) The levels of Ku80 in each region correlates with the amount of DNA damage in the region. STR and HIP express a similar level of Ku80 (a), but DSBs as indicated by the γH2AX marker is 2-5-fold higher in the HIP relative to the STR (b). N=4 male C57BL/6J mice were used to generate each culture, and data collected from n=3 cultures analysis of DSB formation exceeded the DSBR capacity. Data are displayed as a box and whisker plot as in (a). Statistical significance is derived from a 1-way ANOVA, ***P< 0.0001, ****P<0.00001.

Supplementary Fig: 8. No DSB markers are detected in NIH 3T3 cells without peroxide treatment or without transfection of the Cas9Guide RNAs. (a) Negative control for DSBs due to peroxide treatment, as described in Fig. 9a-c for NIH3T3 cells. Cell were cultured for 30 minutes in the absence of peroxide before washing, fixation and staining with antibodies to γH2AX (green) and 53BP1 (red). Untreated cells did not stain with DSBs markers. (b) Negative control for Cas9D10A and Cas9 production of DSBs as described in experiments 2 and 3 in Fig. 9 for NIH3T3 cells. No DSB markers (γH2AX (green) or 53BP1 (red)) were detected in NIH3T3 cells cultured without transfection of either cas9 or Cas9D10A.(a ,b, left panel). The sgMajSat* RNA expression vector was transfected as a measure of cell integrity and is represented as gray.

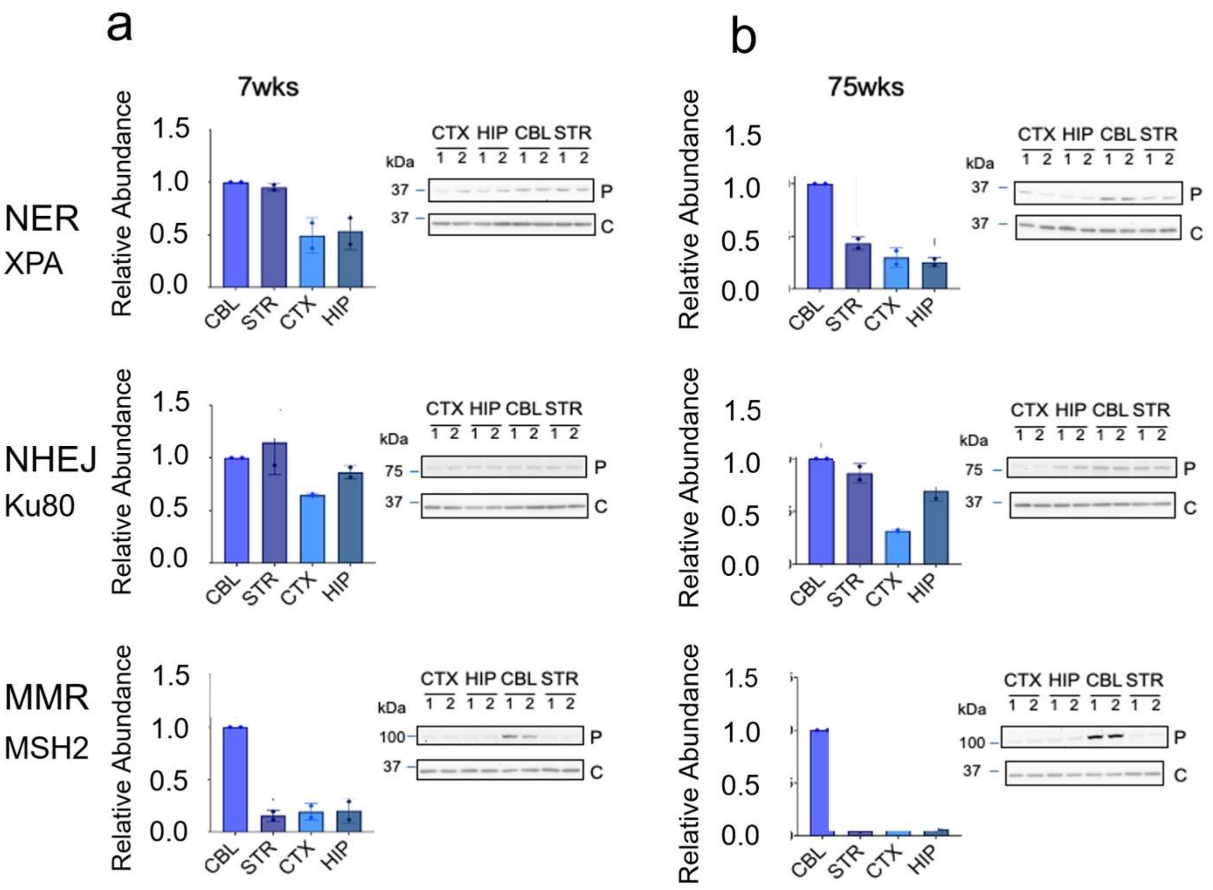

a
b
7wks
75wks
NER
XPA
NHEJ
Ku80
MMR
MSH2
Relative Abundance
1.5
1.0
0.5
0.0
CBL
STR
CTX
HIP
CTX HIP CBL STR
1 2 1 2 1 2 1 2
kDa
37
75
100
P
C

Supp. Fig 1

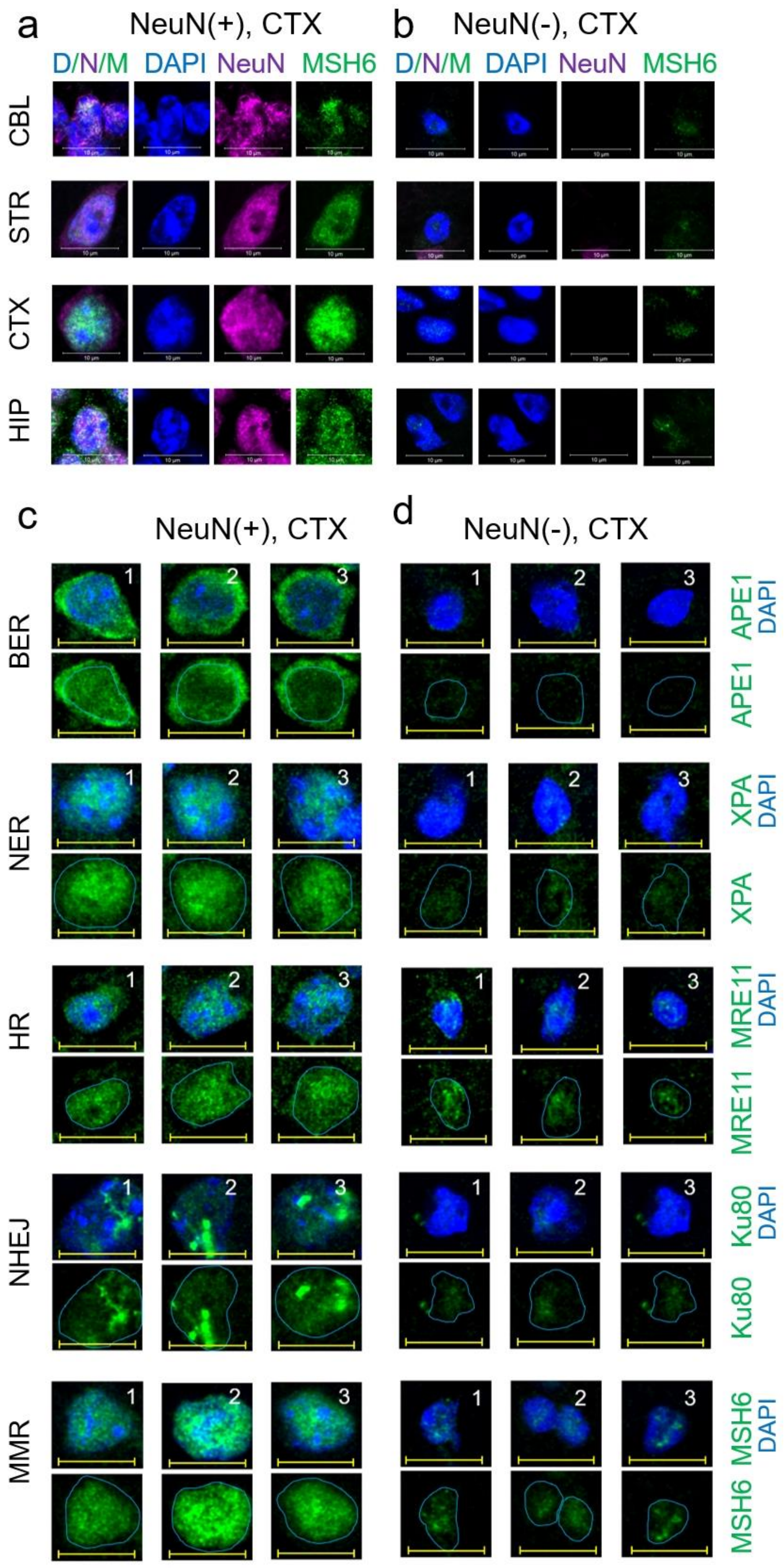
a
NeuN(+), CTX
b
NeuN(-), CTX
D/N/M
DAPI
NeuN
MSH6
CBL
STR
CTX
HIP
c
NeuN(+), CTX
d
NeuN(-), CTX
BER
NER
HR
NHEJ
MMR
APE1 DAPI
APE1
XPA DAPI
XPA
MRE11 DAPI
MRE11
Ku80 DAPI
Ku80
MSH6 DAPI
MSH6

Supp. Fig 2

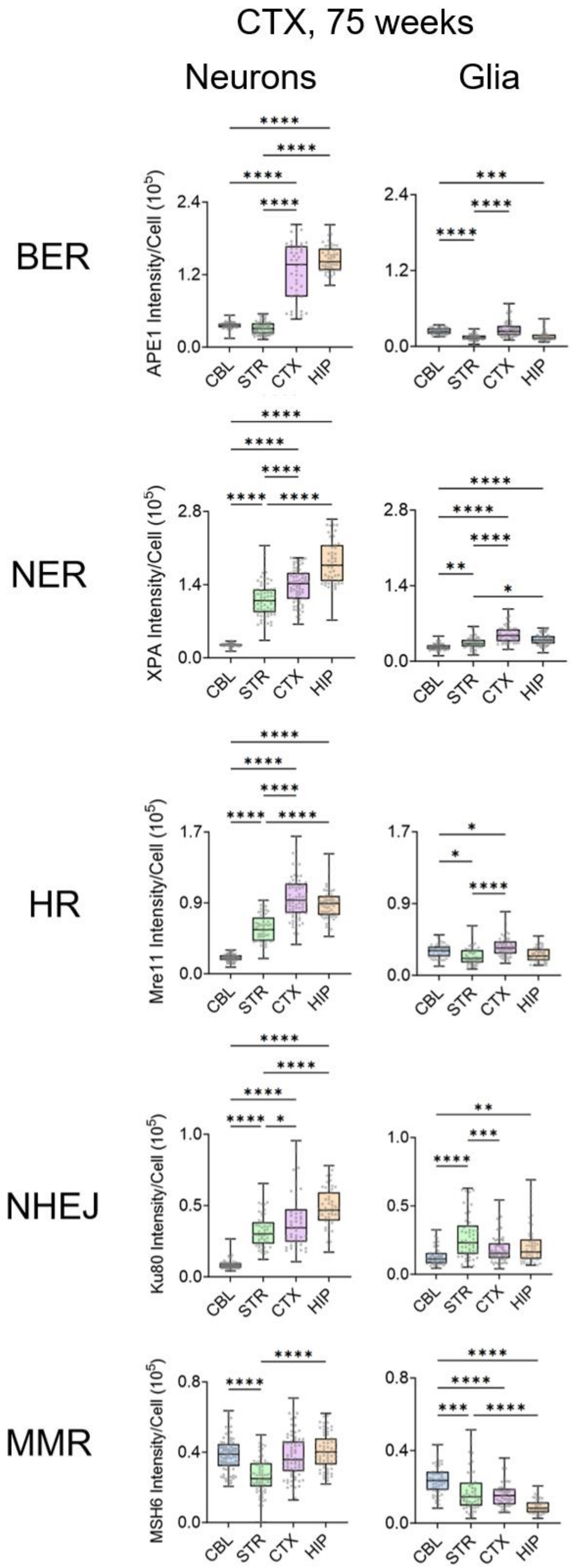
CTX, 75 weeks
Neurons
Glia
BER
NER
HR
NHEJ
MMR
APE1 Intensity/Cell (10^5)
XPA Intensity/Cell (10^5)
Mre11 Intensity/Cell (10^5)
Ku80 Intensity/Cell (10^5)
MSH6 Intensity/Cell (10^5)
CBL
STR
CTX
HIP

Supp. Fig 3

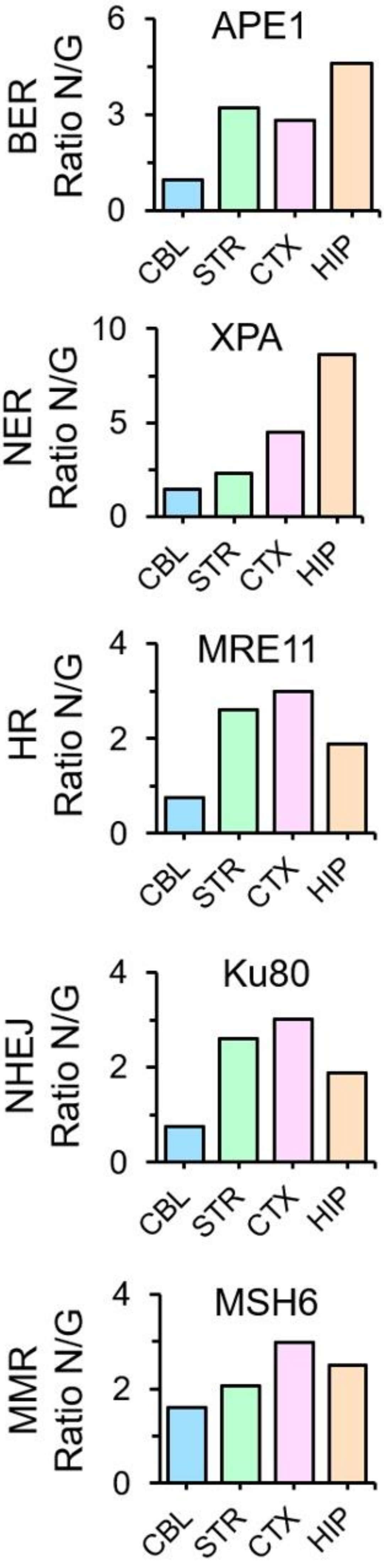
APE1
BER
Ratio N/G
6
3
0
CBL
STR
CTX
HIP
XPA
NER
Ratio N/G
10
5
0
CBL
STR
CTX
HIP
MRE11
HR
Ratio N/G
4
2
0
CBL
STR
CTX
HIP
Ku80
NHEJ
Ratio N/G
4
2
0
CBL
STR
CTX
HIP
MSH6
MMR
Ratio N/G
4
2
0
CBL
STR
CTX
HIP

Supp. Fig 4

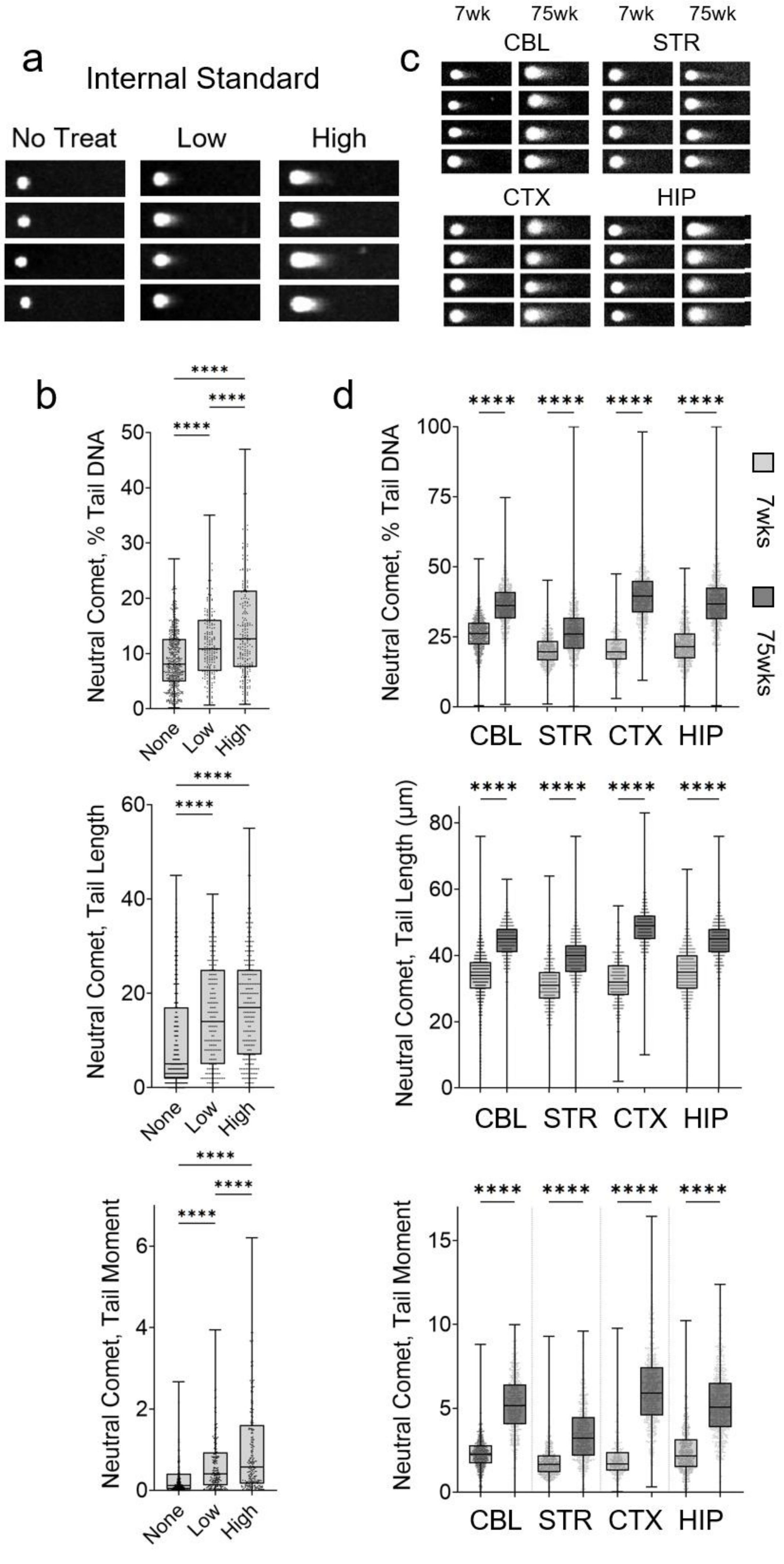
a
Internal Standard
No Treat
Low
High
c
7wk
75wk
7wk
75wk
CBL
STR
CTX
HIP
b
Neutral Comet, % Tail DNA
None
Low
High
Neutral Comet, Tail Length
Neutral Comet, Tail Moment
d
Neutral Comet, % Tail DNA
Neutral Comet, Tail Length (µm)
Neutral Comet, Tail Moment
CBL
STR
CTX
HIP
7wks
75wks
****

Supp. Fig 5

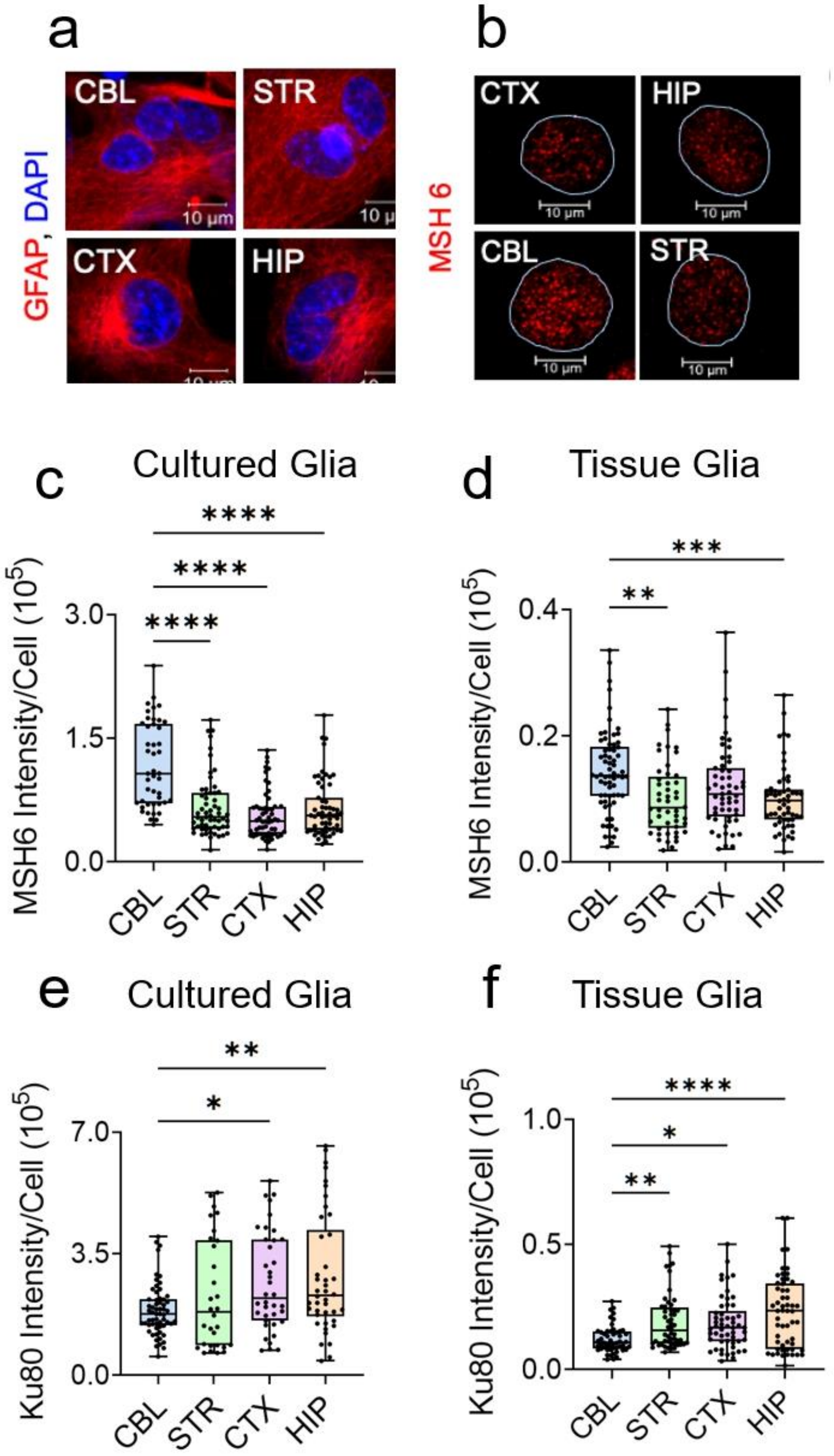

a
CBL
STR
CTX
HIP
GFAP, DAPI
10 µm
b
MSH 6
CTX
HIP
CBL
STR
10 µm
c
Cultured Glia
MSH6 Intensity/Cell (10^5)
3.0
1.5
0.0
CBL
STR
CTX
HIP
****
****
****
d
Tissue Glia
MSH6 Intensity/Cell (10^5)
0.4
0.2
0.0
CBL
STR
CTX
HIP
***
**
e
Cultured Glia
Ku80 Intensity/Cell (10^5)
7.0
3.5
0.0
CBL
STR
CTX
HIP
**
*
f
Tissue Glia
Ku80 Intensity/Cell (10^5)
1.0
0.5
0.0
CBL
STR
CTX
HIP
****
*
**

Supp. Fig 6

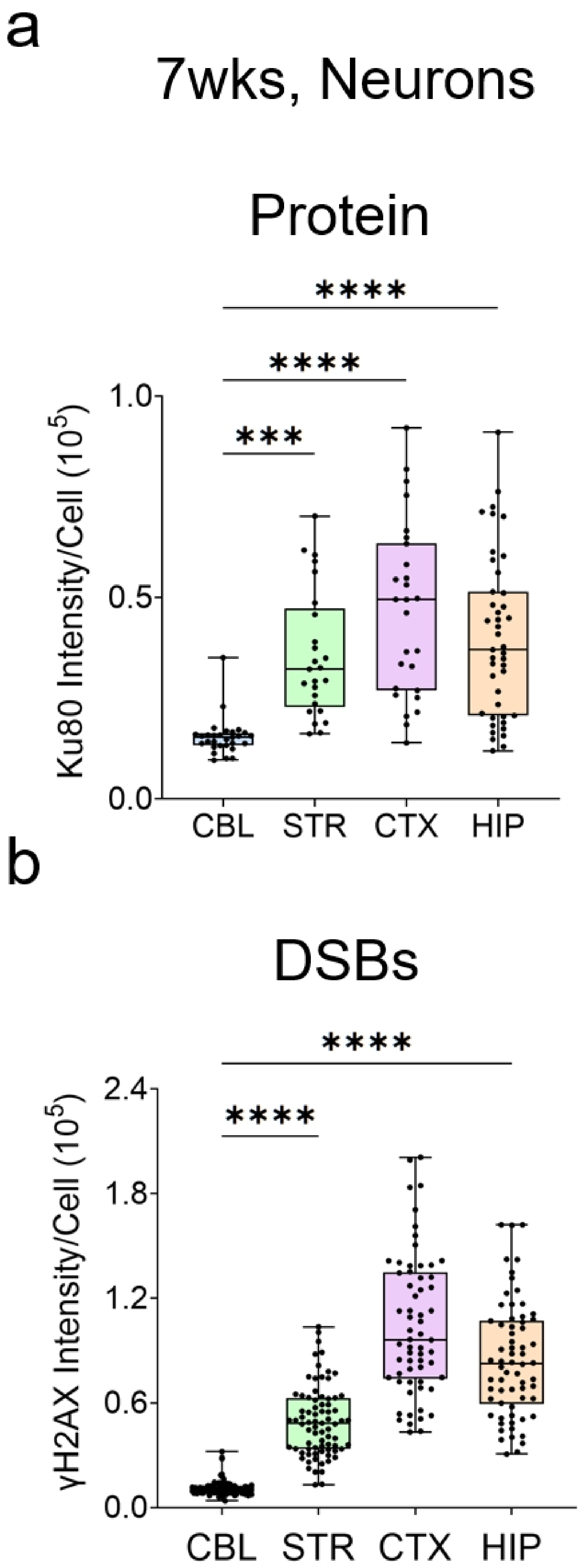
a
7wks, Neurons
Protein
****
****
***
Ku80 Intensity/Cell (10^5)
1.0
0.5
0.0
CBL
STR
CTX
HIP
b
DSBs
****
****
γH2AX Intensity/Cell (10^5)
2.4
1.8
1.2
0.6
0.0
CBL
STR
CTX
HIP

Supp. Fig 7

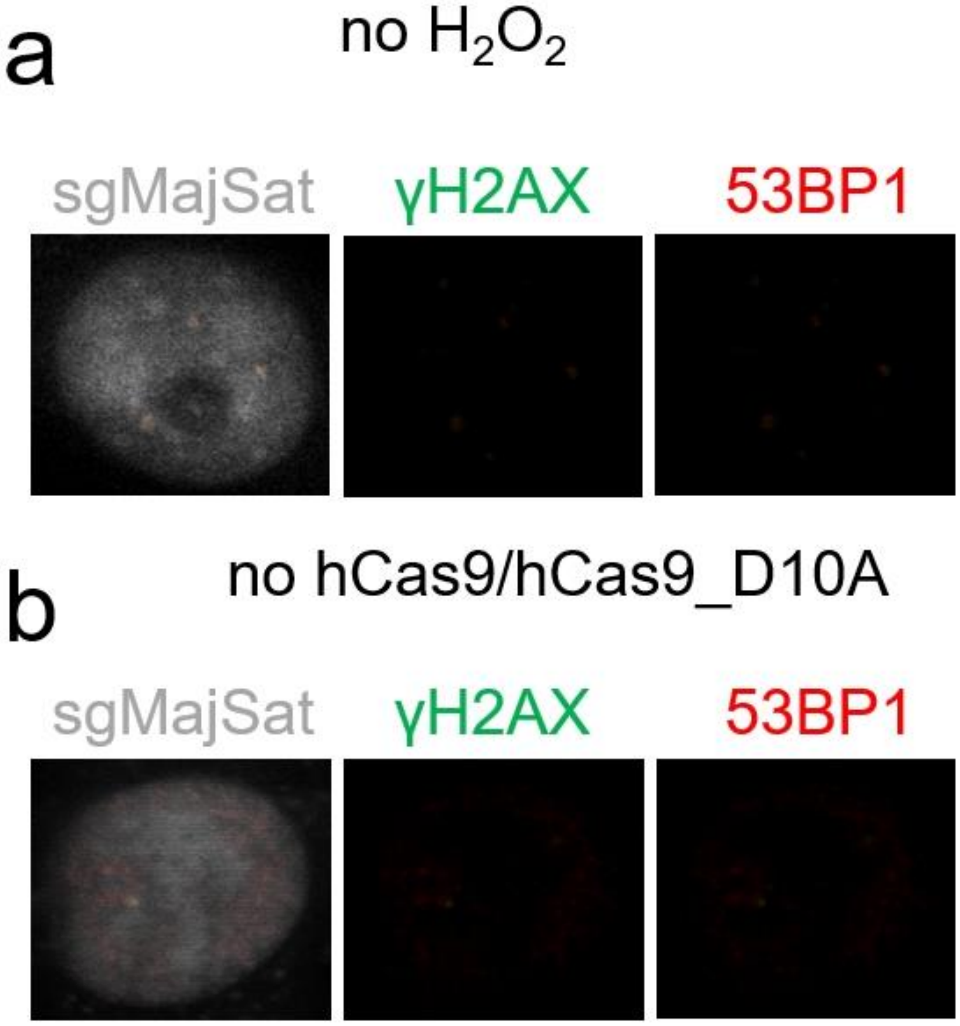
a
no H_2O_2
sgMajSat
γH2AX
53BP1
b
no hCas9/hCas9_D10A
sgMajSat
γH2AX
53BP1

Supp. Fig 8

Supplementary Table 1: Probability of regional differences in repair protein expression in Neurons (Neu) and Glia (GL)(in Fig. 3)[a].

Protein	Cell type	CBL v. STR	CBL v. CTX	CBL v HIP	STR v CTX	STR v HIP	CTX v. HIP
APE	Neu	2.12E-03	8.77E-21	5.24E-46	1.85E-25	1.05E-51	0.12
APE	GL	6.47E-14	0.91	1.31E-03	3.45E-05	0.71	2.90E-04
XPA	Neu	4.86E-30	4.48E-43	9.43E-48	3.19E-02	2.10E-09	2.09E-06
XPA	GL	9.11E-03	7.12E-09	7.82E-11	9.24E-04	0.51	2.08E-03
MRE11	Neu	1.55E-29	9.86E-29	4.86E-23	1.19E-05	3.54E-12	7.12E-18
MRE11	GL	0.15	3.74E-06	4.34E-06	3.39E-03	8.85E-03	1.84E-08
Ku80	Neu	8.64E-24	4.41E-22	4.39E-21	7.15E-03	0.33	0.07
Ku80	GL	6.73E-05	1.52E-04	3.66E-09	0.74	0.14	0.07
MSH6	Neu	4.09E-03	1.84E-08	0.18	2.67E-12	1.30E-03	1.66E-04
MSH6	GL	3.41E-04	0.20	7.44E-03	0.07	0.63	0.16

a. Statistical probability of variance (*P*) calculated using 2-tailed homoscedastic t-test. n = 50 cells of each type (Neu) Neuron and (GL) Glia analyzed for each brain region.

Supplementary Table 2: Key Resources.

Reagent or Resource		Source	Catalogue ID
**Antibody**			
Mouse anti-NeuN alexafluor-488 conjugate	1:500	EMD Millipore	MAB377X
Mouse anti-GFAP Cy3 conjugate	1:500	Abcam	ab49874
Mouse anti-APE1	1:500	Novus	13B8E5C2
Mouse anti-Ku80		Santa Cruz	515736
Mouse anti-ERCC1		Santa Cruz	17809
Rabbit anti-MSH2		Abcam	ab92471
Mouse anti-MSH3		EMD Millipore	MABE324
Rabbit anti-MSH6		Abcam	Ab92471
Rabbit anti-XPA		AbClonal	A1626
Rabbit anti-MRE11		Novus	NB100-142
Donkey anti-Mouse alexafluor-488 conjugate		Jackson Immunores.	715-545-150
Goat anti-Mouse alexafluor-568 conjugate		Invitrogen	A21124
Donkey anti-Rabbit alexafluor-488 conjugate		Jackson Immunores.	711-545-152
Goat anti-Rabbit alexafluor-555 conjugate		Invitrogen	A32732
**Chemicals, peptides, and recombinant proteins**			
Fc Receptor Block		Innovex	NB309
Background Buster		Innovex	NB306
Tissue-Tek O.C.T. Compound		Sakura	4583
TrueBlack		Biotium	23007
Monarch RNase A		New England Biolabs	T3018L
ImmuMount		Epredia	9990402
Nuclease P1		New England Biolabs	M0660
Quick Calf Alkaline Phosphatase		New England Biolabs	M0525
hCas9D10A nickase		Addgene	#41816
hCas 9 is pCDNA3.3TOPO-T7-hCas9		Addgene	#161876
T-PER Tissue Protein Extraction Reagent		Thermo Scientific	78510
Halt protease inhibitor cocktail		Thermo Scientific	78420
Pierce 660 Protein Assay reagent		Thermo Scientific	22660
NuPAGE Sample Reducing Agent		Invitrogen	NP0009
Novex WedgeWell		Thermo Fisher	XP04205BOX
Amersham ECL Western Blotting Reagent		Sigma-Aldrich	GERPN2235

**Critical commercial assays**			
DNA/RNA Oxidation Assay		Cayman chemicals	589320
DNeasy Blood and Tissue Kit		Qiagen	69504
**Experimental models: Organisms/strains**			
C57Bl/6J male mice		Jackson Labs	000664
NIH/3T3 fibroblasts		ATCC	CRL-1658
**Software and algorithms**			
ImageJ:Fiji		imagej.net/software/fiji/	Version 2.15.0
Prism Graph Pad		www.graphpad.com/features	version 9.5.1
Trivagen (comet analysis)		R&D	v1
VersaDoc MP 4000 Imaging System		Biorad	Quantity One 1-D
Image Lab software		Biorad	V1

Supplementary Table 3: Antibodies tested.

DNA Repair Pathway	Protein	Antibody Host Species	Vendor	Catalog Number	Worked? (Mouse brain tissue lysates)	Worked? (Mouse astrocyte P1 culture lysates)
Homologous Recombination (HR)	MRE11	Rb	Novus Biologicals	NB100-142	Yes	
Non-homologous End Joining (NHEJ)	Ku70	Ms	Invitrogen	MA5-13110	No	No
		Ms	SCBT	sc-17789	No	
		Rb	CST	4588S	Yes	Yes
	Ku80	Rb	Thermo Fisher	PA5-17454	many bands	
		Ms	SCBT	sc-515736	Yes	Yes
Base Excision Repair (BER)	APE1	Ms	Novus Biologicals	NB100-116SS	Yes	
Nucleotide Excision Repair (NER)	ERCC1	Ms	SCBT	sc-17809	Yes	Yes
	XPA	Rb	ABclonal	A1626	Yes	
Mismatch Repair (MMR)	MSH2	Rb	Abcam	ab92473	Yes	Yes
	MSH6	Rb	Abcam	ab92471	Yes	Yes
