# Supplementary material for: Base excision repair and double strand break repair cooperate to modulate the formation of unrepaired double strand breaks in mouse brain
Source: Nat Commun. 2024 Sep 4;15:7726. doi: 10.1038/s41467-024-51906-5 (PMC11375129; doi:10.1038/s41467-024-51906-5)
Supplement: Supplementary file 4 — Source data [file 41467_2024_51906_MOESM4_ESM.zip › Supplementary Source File data for NCOMMS-23-38584B_7-15-24.pdf]

APE1

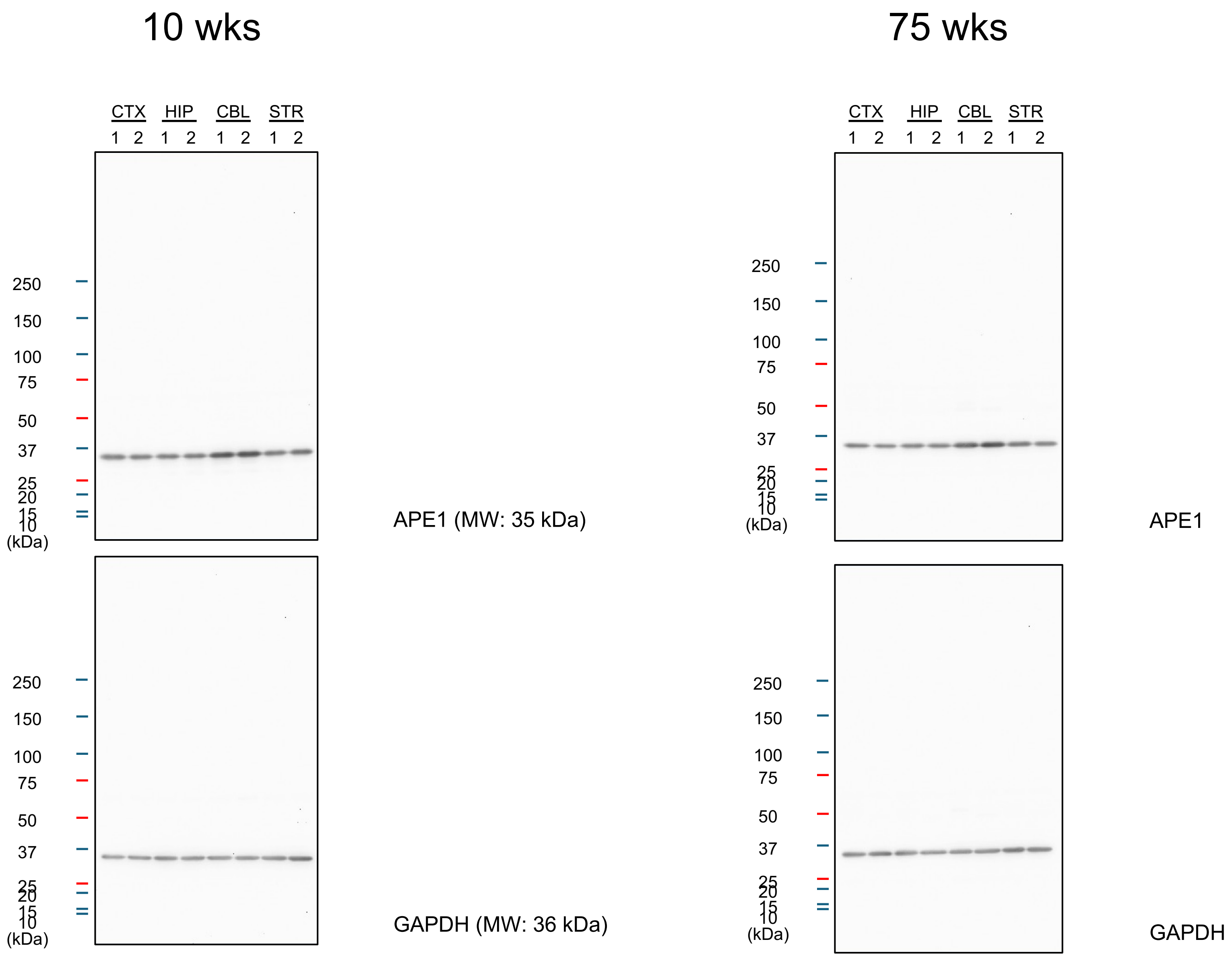
10 wks
75 wks
CTX
HIP
CBL
STR
1 2 1 2 1 2 1 2
250
150
100
75
50
37
25
20
15
10
(kDa)
APE1 (MW: 35 kDa)
GAPDH (MW: 36 kDa)
APE1
GAPDH

ERCC1

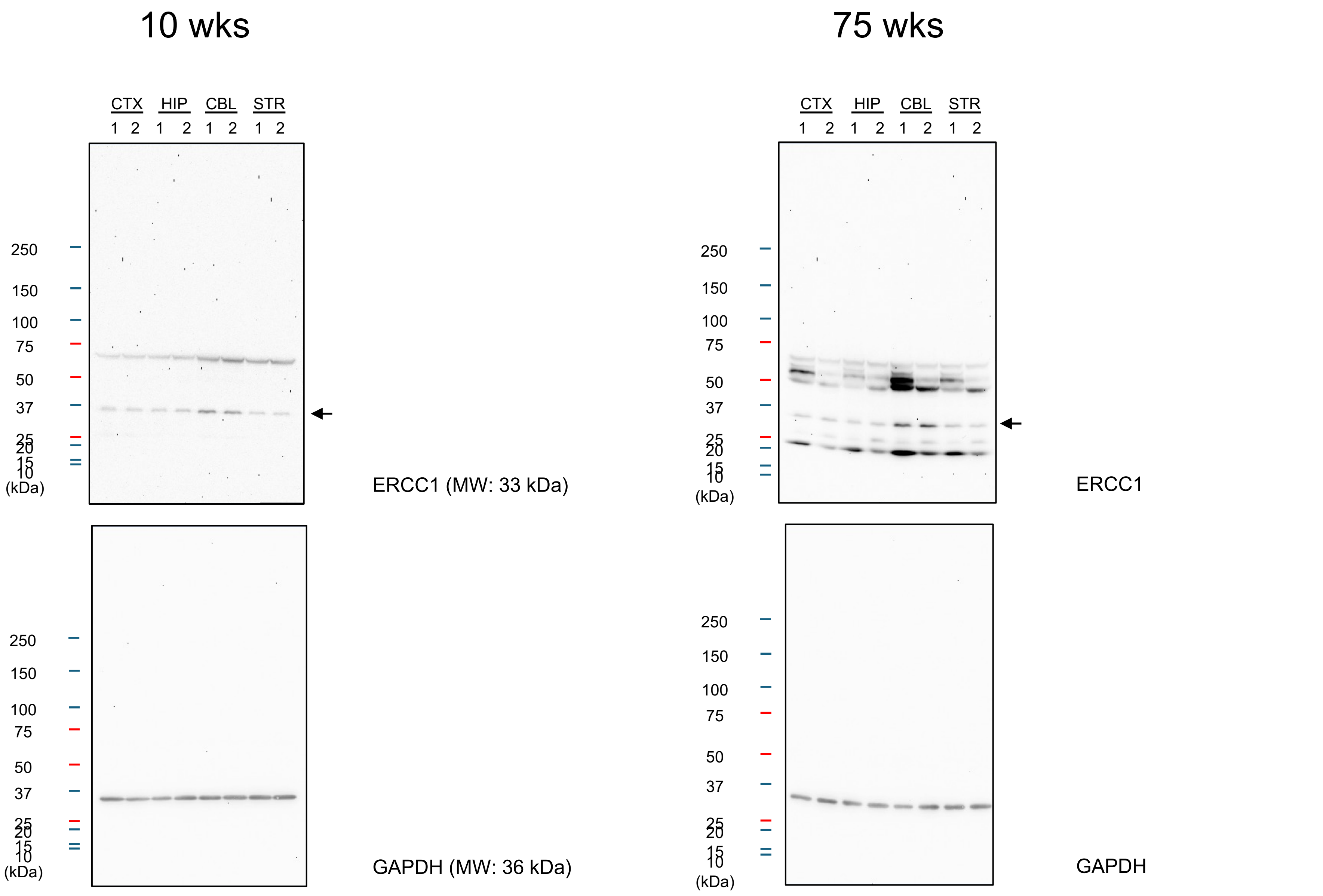
10 wks
75 wks
CTX HIP CBL STR
1 2 1 2 1 2 1 2
250
150
100
75
50
37
25
20
15
10
(kDa)
ERCC1 (MW: 33 kDa)
GAPDH (MW: 36 kDa)
ERCC1
GAPDH

MRE11

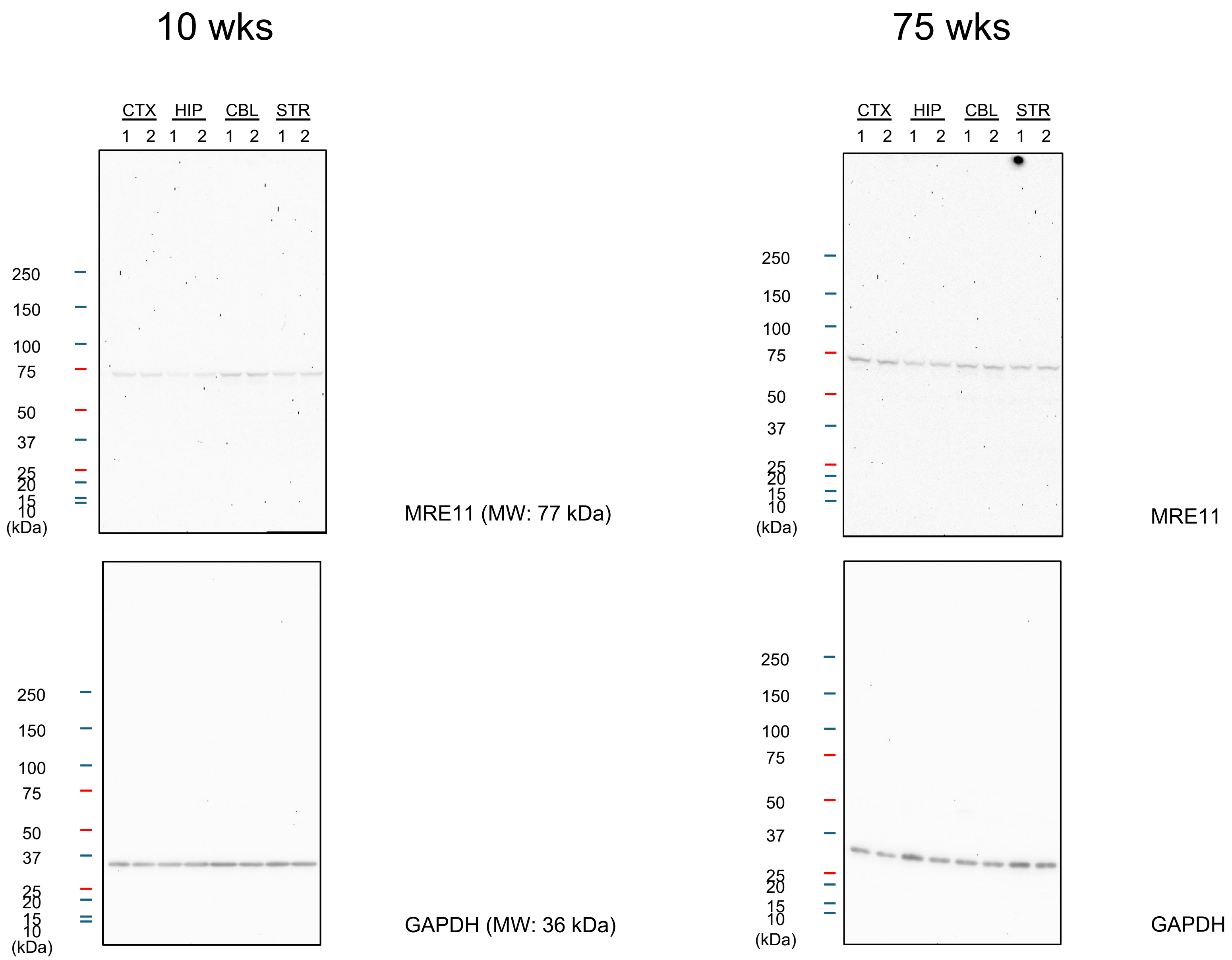
10 wks
75 wks
CTX HIP CBL STR
1 2 1 2 1 2 1 2
250
150
100
75
50
37
25
20
15
10
(kDa)
MRE11 (MW: 77 kDa)
GAPDH (MW: 36 kDa)
MRE11
GAPDH

Ku70

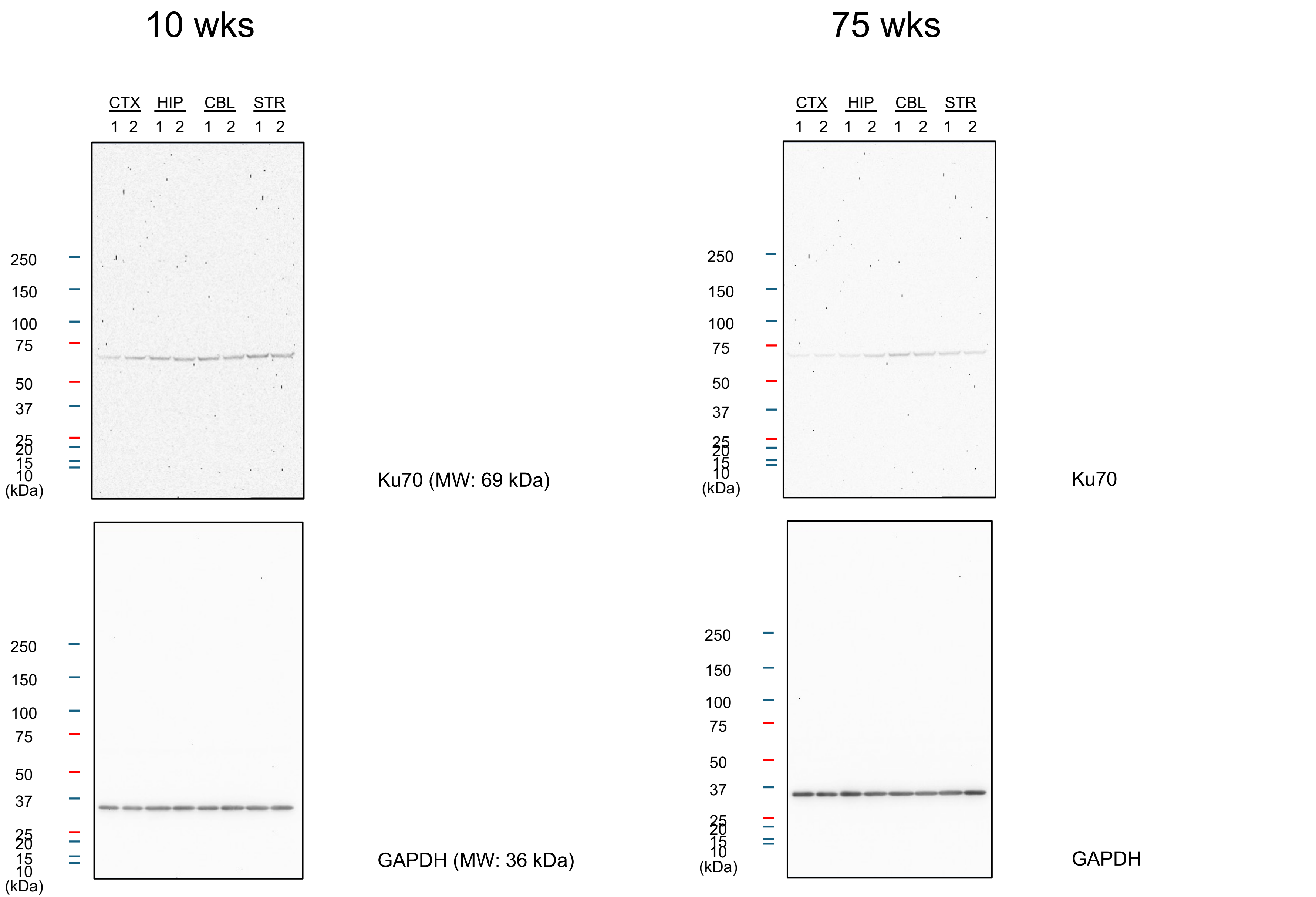

10 wks
75 wks
CTX
HIP
CBL
STR
1 2 1 2 1 2 1 2
250
150
100
75
50
37
25
20
15
10
(kDa)
Ku70 (MW: 69 kDa)
GAPDH (MW: 36 kDa)
Ku70
GAPDH

MSH6

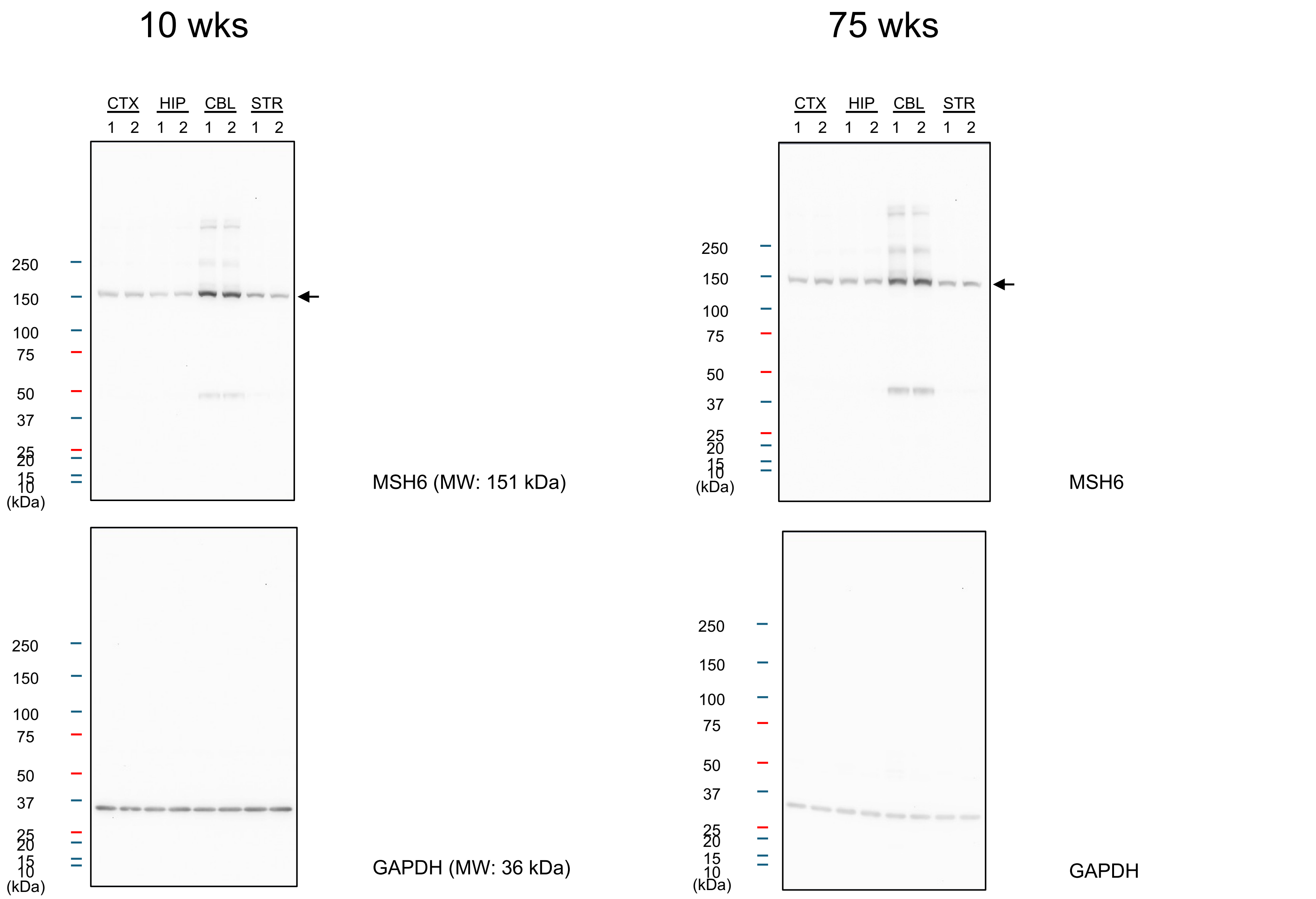

10 wks
75 wks
CTX HIP CBL STR
1 2 1 2 1 2 1 2
250
150
100
75
50
37
25
20
15
10
(kDa)
MSH6 (MW: 151 kDa)
MSH6
GAPDH (MW: 36 kDa)
GAPDH

XPA

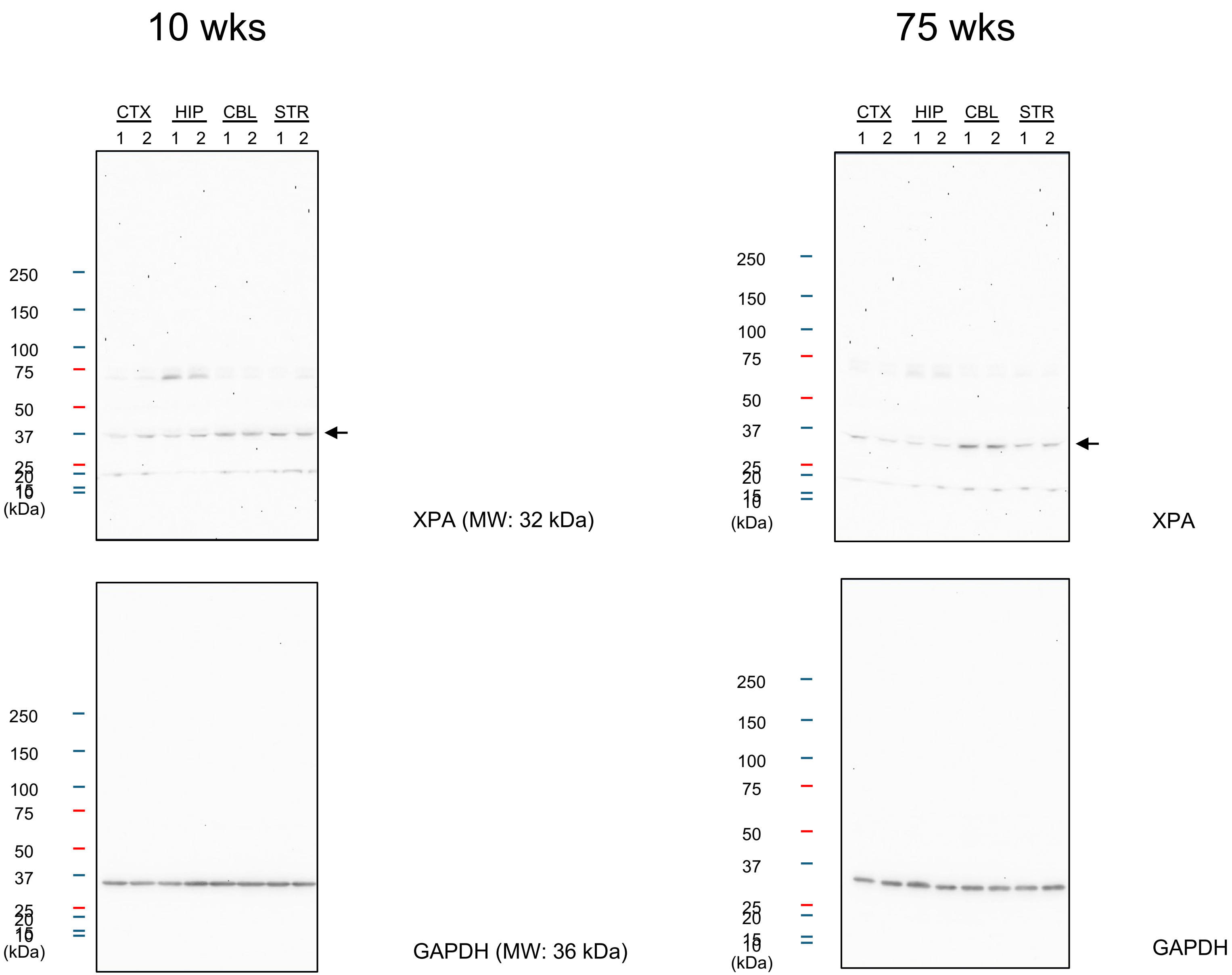
10 wks
75 wks
CTX HIP CBL STR
1 2 1 2 1 2 1 2
250
150
100
75
50
37
25
20
15
10
(kDa)
XPA (MW: 32 kDa)
GAPDH (MW: 36 kDa)
XPA
GAPDH

Ku80

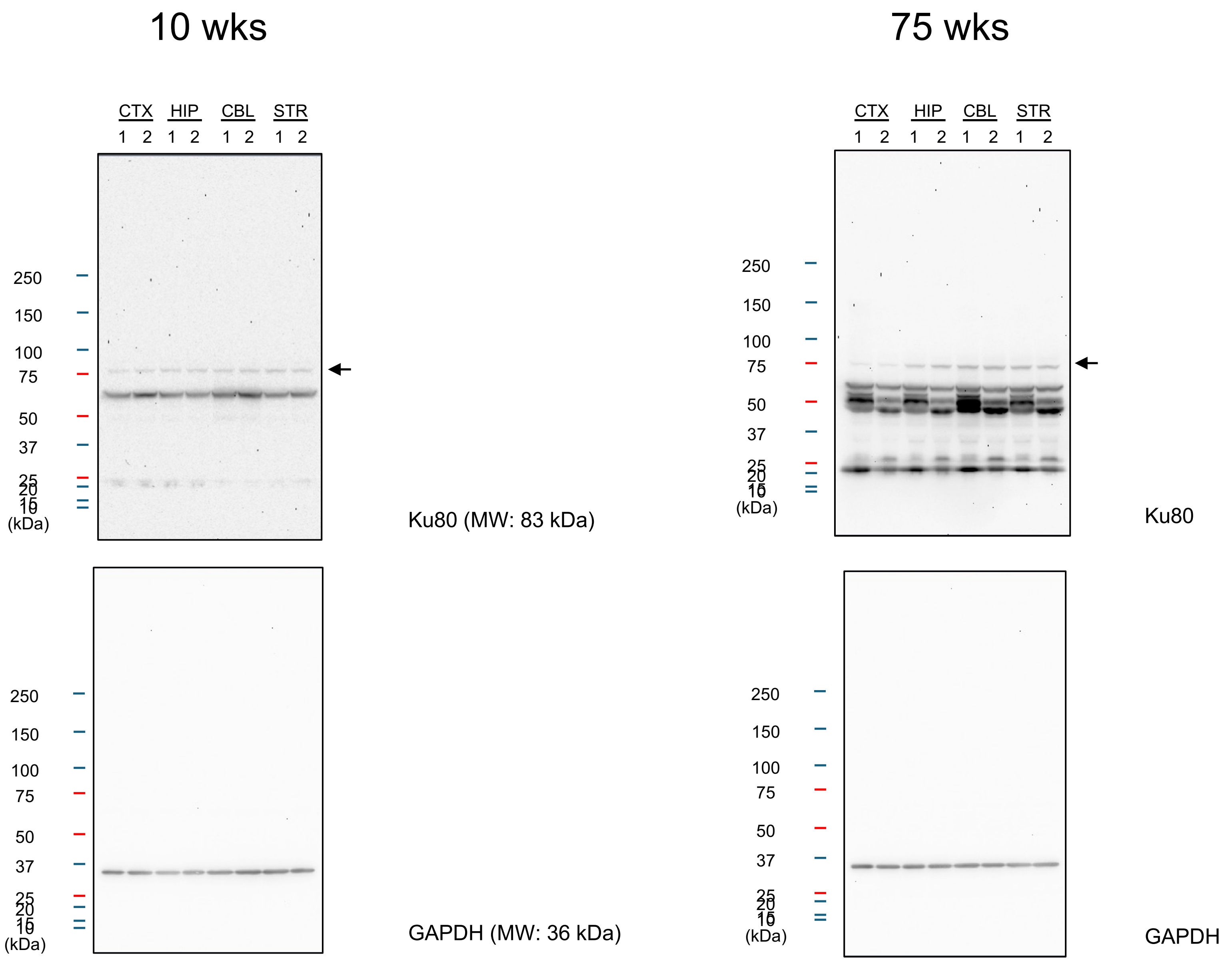
10 wks
75 wks
CTX HIP CBL STR
1 2 1 2 1 2 1 2
250
150
100
75
50
37
25
20
15
10
(kDa)
Ku80 (MW: 83 kDa)
GAPDH (MW: 36 kDa)
Ku80
GAPDH

MSH2

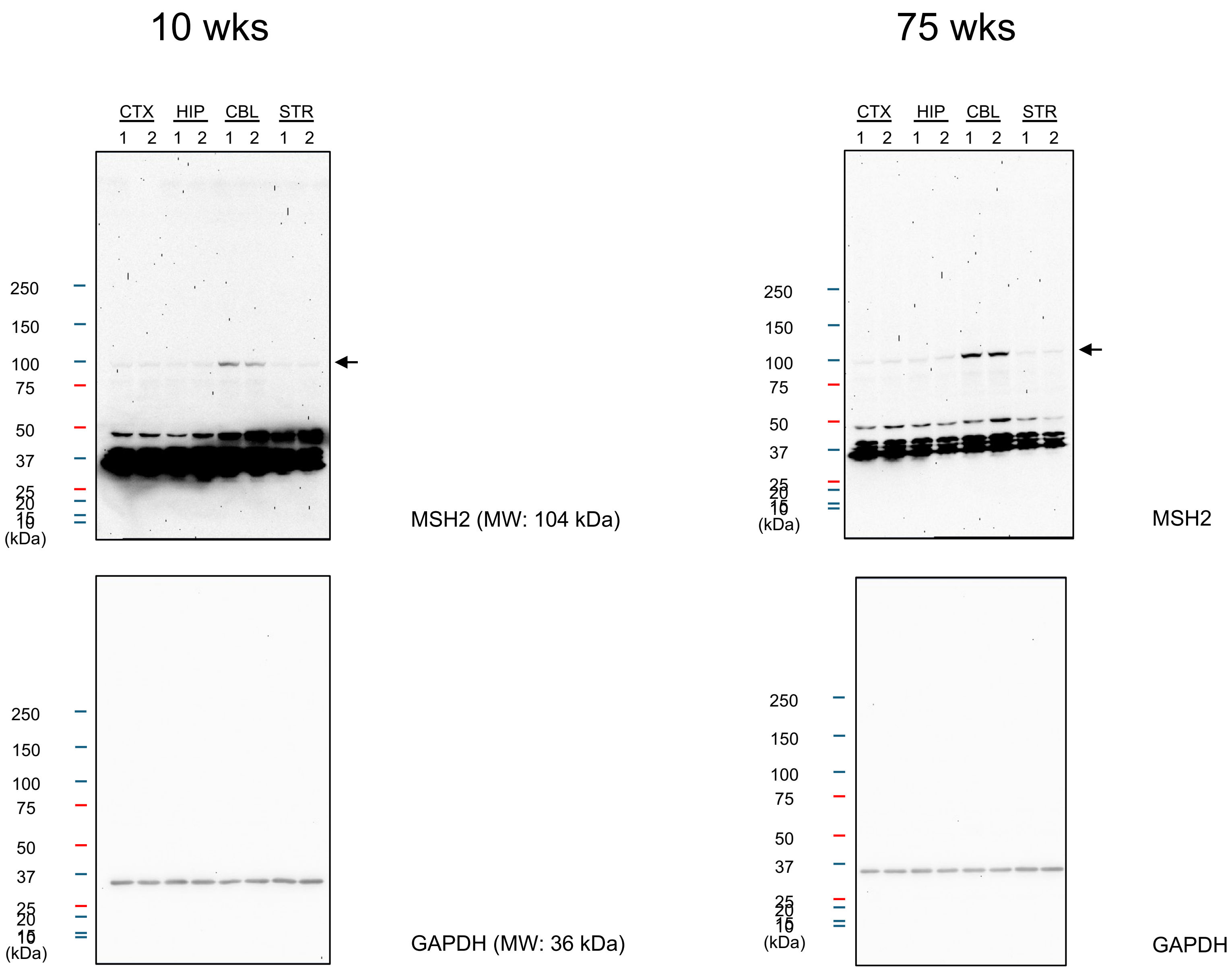
10 wks
75 wks
CTX
HIP
CBL
STR
1 2 1 2 1 2 1 2
250
150
100
75
50
37
25
20
15
10
(kDa)
MSH2 (MW: 104 kDa)
GAPDH (MW: 36 kDa)
MSH2
GAPDH

Supplementary information Table 3: Antibody testing

DNA Repair Pathway	Protein	WB Tried?	Antibodies Tried (Species)	Vendor	Catalog Number	Worked? (Mouse brain tissue lysates)	Worked? (Mouse astrocyte P1 culture lysates)
Homologous Recombination (HR)	BRCA1	Yes	Ms	Novus Biologicals	MAB22101		No
	CtIP	Yes	Ms	Active Motif	61141	No	No
	BRCA2	No					
Non-homologous End Joining (NHEJ)	Ku70	Yes	Ms	Invitrogen	MA5-13110	No	No
			Ms	SCBT	sc-17789	No	
			Rb	CST	4588S		Yes
	Ku80	Yes	Rb	Thermo Fisher	PA5-17454	many bands	
			Ms	SCBT	sc-515736	Yes	Yes
	DNA-PK	Yes	Rb	Abcam	ab32566	150 kDa band	150 kDa +
	Artemis	Yes	Ms	Bethyl Laboratories	A304-902A-M	many bands	50 kDa
Base Excision Repair (BER)	APE1	Yes	Ms	Novus Biologicals	13B8E5C2	Yes	
	OGG1	Yes	Rb	Novus Biologicals	NB100-106	Many bands	
	PARP-1	Yes	Ms	Bio-Rad	MCA1522G	No	
Nucleotide Excision Repair (NER)	ERCC1	Yes	Ms	SCBT	sc-17809	Yes	Yes
	XPF	Yes	Ms	SCBT	sc-136153	No	No
	XPG	No					
Mismatch Repair (MMR)	MSH2	Yes	Rb	Abcam	ab92473	Yes	Yes
	MSH3	Yes	Ms	BD Biosciences	611390	No	
			Rb	Thermo Fisher	PA5-75306	No	
			Ms	EMD Millipore	MABE324	Yes	Yes
	MSH6	Yes	Rb	Abcam	ab92471	Yes	Yes
